# Effectiveness of lopinavir/ritonavir on COVID-19-related pneumonia in a child with COVID-19-associated Kawasaki disease

**DOI:** 10.1017/S1047951120004291

**Published:** 2020-11-13

**Authors:** Zerrin Orbak, Fuat Laloglu, Hulya Akat

**Affiliations:** Department of Pediatrics, Ataturk University, Erzurum, Turkey

**Keywords:** Kawasaki disease, COVID-19, pneumonia

## Abstract

The large outbreak of coronavirus disease 2019 (COVID-19) is spreading all over the world rapidly. There have recently been publications in the literature regarding the relationship between COVID-19 and Kawasaki disease, but there is no sufficient knowledge about the treatment and follow-up.

Here, a paediatric case with Kawasaki disease associated with 2019 novel coronavirus infection and successful treatment of COVID-19-related pneumonia with lopinavir/ritonavir is reported.

Kawasaki disease (previously called mucocutaneous lymph node syndrome) is one of the most common vasculitides of childhood. Although it is typically a self-limited condition, complications such as coronary artery aneurysms, arrhythmias, depressed myocardial contractility, heart failure, and peripheral arterial occlusion may develop and cause mortality and significant morbidity. In addition to these problems, hypoalbuminaemia, electrolyte imbalance, and hydrops of the gallbladder were observed.^1^ Recently, a severe form of Kawasaki disease presenting with haemodynamic instability and shock has been reported, and it is called Kawasaki disease shock syndrome. This form has been associated with more severe markers of inflammation. The aetiology of Kawasaki disease has not been fully understood although various studies represented that viruses such as adenovirus and coronavirus have been shown in patients with Kawasaki disease.^1^ Coronaviruses may cause diseases ranging from common cold illnesses to more severe diseases such as Severe Acute Respiratory Syndrome and Middle East Respiratory Syndrome. There are the pandemic with the emergence and spread of 2019 novel coronavirus or the severe acute respiratory syndrome coronavirus 2.^2^ There have recently been publications in the literature regarding the relationship between COVID-19 and Kawasaki disease^3^, but there is no sufficient knowledge about the treatment and follow-up.

Here, the case with Kawasaki disease associated with 2019 novel coronavirus infection and successful treatment of pneumonia with lopinavir/ritonavir were reported.

## Case report

A 10-year-old healthy boy previously presented to our hospital with a 6-day history of high-grade fever, non-productive cough, anorexia, headache, and malaise. He also complained of bilateral non-purulent bulbar conjunctivitis, lip erythema, cervical lymphadenitis on the right side, oedema of hands and feet, and maculopapular skin rash one day before admission to the hospital.

From his history, it was learned that his parents (both of them) were diagnosed with COVID-19 10 days before he got sick, and isolation at home was recommended.

**Figure 1.**
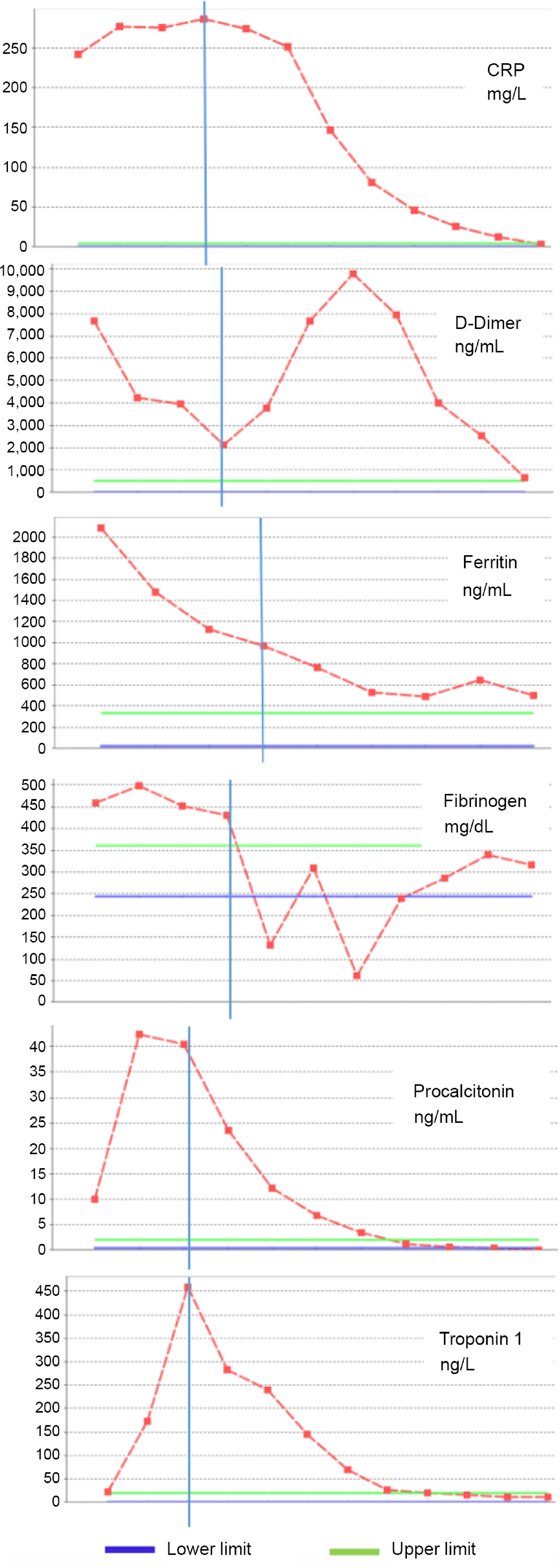
The course of the inflammatory parameters (the vertical blue line shows the beginning of lopinavir/ritonavir treatment).

**Figure 2.**
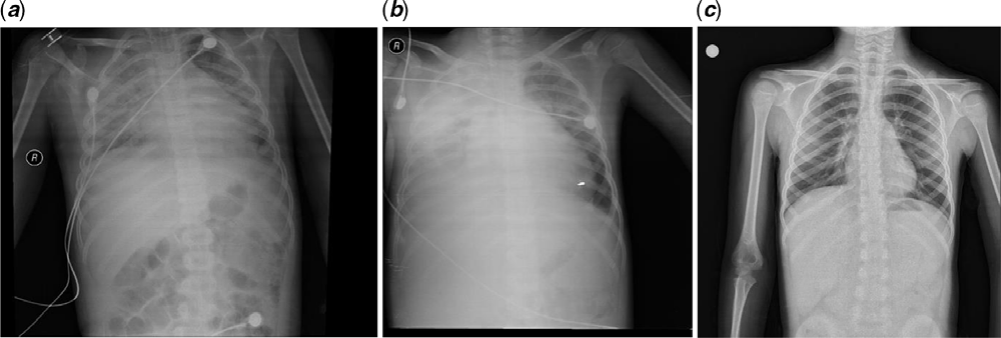
Chest X-ray radiographs (a posteroanterior radiograph of the chest in the upright position of the patient): from left to right. (***a***) admission to the hospital, (***b***) at the beginning of treatment, (***c***) after 14 days of treatment.

**Figure 3.**
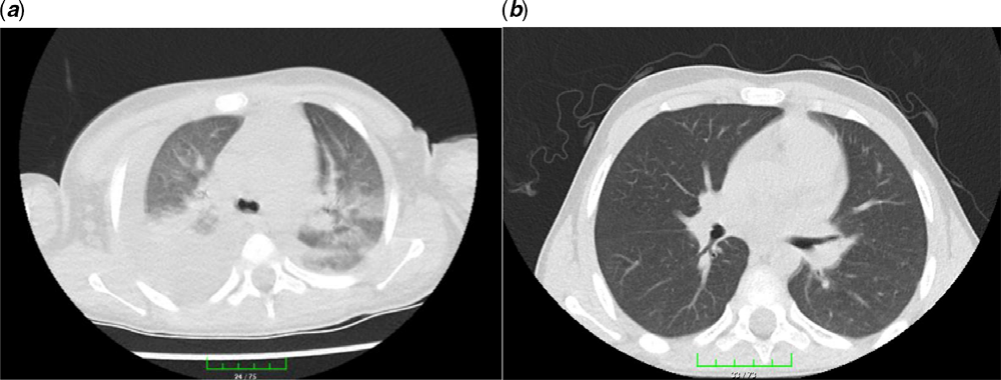
Chest high-resolution CT: from left to right. (***a***) Patchy or nodular consolidations with peripheral ground-glass opacities in subpleural areas and (***b***) resolution after 14 days of lopinavir/ritonavir therapy.

On admission, he was not dyspnoeic with a body temperature of 39.2°C. Nasopharyngeal/oropharyngeal swabs (NP/OP) swabs were sent to the reference laboratory and tested negative for 2019 novel coronavirus by a real-time reverse transcriptase polymerase chain reaction assay. Other respiratory pathogens were also negative. Initial laboratory results were as follows: white blood cell count 6040/μL (27.3% lymphocytes), haemoglobin 10.6 g/dL, platelet count 116,000/μL, serum Na 129 mEq/L, and serum albümin 3.1 g/dL. Figure 1 showed the course of inflammatory parameters at the admission and during treatment. His chest radiological imaging (X-ray and high-resolution CT) showed pneumonic infiltrates with predominance on the right (Figs 2 and 3). Echocardiographic study revealed normal left ventricular functions and coronary arteries. Pericardial effusion and mitral regurgitation were not present. It was diagnosed that he had Kawasaki disease and COVID-19 infection because of positive family history and patchy or nodular consolidations with peripheral ground-glass opacities in subpleural areas although negative swap polymerase chain reaction result. Treatment was started with ampicillin-sulbactam (250 mg/kg/day ampicillin; sulbactam IV divided every 6 hours), azithromycin (15 mg/kg/day), oseltamivir (60 mg twice daily), and hydroxychloroquine (10 mg/kg/initial dose; 6 mg/kg twice daily). Two main treatments for Kawasaki disease are aspirin and intravenous immune globulin. Because aspirin may cause side effects, including Reye’s syndrome, clexan (100 IU/kg/dose BD) was added to reduce blood clots. After the first intravenous immune globulin dose (2 gr/kg/dose), his fever did not improve. So, the second dose of intravenous immune globulin was given. The serologic test for the presence of IgM and IgG antibodies in plasma against COVID-19 was weakly positive. On hospital day 4, his fever decreased but pneumonia progressed (Figs 2 and 3). He had dyspnoea and complained of a nonproductive cough. SpO2 decreased to 75%. He needed high-flow nasal canula oxygen therapy, but not intubation. Oseltamivir and hydroxychloroquine were stopped and lopinavir/ritonavir (300/75 mg/day) was started. Azithromycin therapy was stopped on hospital day 5. Treatment with ampicillin-sulbactam was continued for 10 days.

On hospital day 7, his clinical condition was improving. Although OP swab was sent again and tested negative for 2019 novel coronavirus by polymerase chain reaction, the serologic test (IgM/IgG antibodies) in plasma against COVID-19 was a strong positive.

Lopinavir/ritonavir treatment was continued for 14 days. He was discharged with just acetylsalicylic acid therapy (150 mg/day) on hospital day 18. His overall clinical condition improved, with increasing appetite, and he was afebrile. During hospitalisation, repeated echocardiographic examinations were done and no new coronary pathology developed. On lopinavir/ritonavir treatment 14, the radiological improvement was complete (Figs 2 and 3). The serologic test (IgM/IgG antibodies) in plasma against COVID-19 was continuingly strong positive.

## Discussion

The diagnosis of Kawasaki disease is based on the last updated guideline of American Heart Association.^4^ Classic Kawasaki disease is diagnosed in the presence of fever for at least 5 days (the day of fever onset is taken to be the first day of fever) together with at least four of the five following principal clinical features: erythema and cracking of lips, strawberry tongue, and/or erythema of oral and pharyngeal mucosa; bilateral bulbar conjunctival injection without exudate; rash: maculopapular, diffuse erythroderma, or erythema multiforme-like; erythema and oedema of the hands and feet in acute phase and/or periungual desquamation in subacute phase; cervical lymphadenopathy (≥1.5 cm diameter), usually unilateral. Our patients’ findings were consistent with classic Kawasaki disease. In our patient triggering infection for typical Kawasaki disease was thought to be COVID-19 infection, because of increasing antibody titers. In case of negative polymerase chain reaction testing, as in our patient, for SARS-CoV-2, antibody testing against COVID-19 infection should be performed in patients who present with features of Kawasaki disease, particularly if there is evidence of COVID-19 exposure.

Thorax CT is a sensitive diagnostic approach in the early period in patients with a negative polymerase chain reaction test for COVID-19.^2^ Ai et al^5^ reported that 94% of patients had bilateral chest findings and they had ground-glass opacities (49%) and consolidation (55%). Also, Zhu et al^6^ showed that 97% of patients had bilateral chest findings and frequency of radiological findings were ground-glass opacities (50%); consolidation (13%); spider web sign (13%); crazy-paving pattern (3%); and pleural effusion (7%). Our patient had consolidation, spider web sign, and patchy or nodular consolidations with peripheral ground-glass opacities.

Could the polymerase chain reaction test for COVID-19 be negative despite of the presence of disease? In the literature, there are cases who had negative NP/OP swabs but positive bronchoalveolar lavage (BAL) fluid.^7^ Because taking BAL fluid is an invasive procedure we recommend that high clinical-radiological suspicion and contact history are important for diagnosis despite a negative NP/OP swabs result. False-negative swab results may be related to the sampling technique, transportation process, and limited gene detection. This condition was reported previously for Severe Acute Respiratory Syndrome and Middle East Respiratory Syndrome outbreaks. So, it can also be explained by the nature of coronavirus. Scientific evidence supported that the target functional receptor of this virus is angiotensin-converting enzyme 2.^8^ It has been shown that surface expression of SARS-CoV-2 receptor, angiotensin-converting enzyme 2, was abundantly on alveolar epithelial cells but negative on the nasal, oral, and nasopharyngeal areas. Also, airways expression of angiotensin-converting enzyme 2 is lower in children than adults.^9^

There was no clear consensus on the treatment of COVID-19. We gave lopinavir/ritonavir treatment for 14 days which completely improved pneumonia. Why did we think that lopinavir/ritonavir treatment could be effective against COVID-19? Both drugs are HIV-1 protease inhibitors and binding well to the SARS-CoV 3C-like protease (SARS-CoV 3CLpro) has been reported.^10^

As a result, we want to share the medical treatment data of our patient with Kawasaki disease associated with COVID-19 infection. Lopinavir/ritonavir treatment is effective in children with pneumonia related to COVID-19 infection. We also think that our findings will provide a basis for paediatricians to obtain early diagnosis and successful treatment.
